# Accuracy of Guided Drilling, Partially Guided Trephination, and Fully Guided Trephination Within a Static Surgical Guide for Apicoectomy in Hard Bone: An In Vitro Study

**DOI:** 10.3390/dj14030155

**Published:** 2026-03-09

**Authors:** Fatima Jasim Humaid Alzaabi, Eszter Nagy, Dániel Gerhard Gryschka, Shishir Ram Shetty, Tarek Elsewify, Gábor Braunitzer, Hatem M. El-Damanhoury, Mark Adam Antal

**Affiliations:** 1 Department of Restorative Dentistry, College of Dental Medicine, University of Sharjah, Sharjah 27272, United Arab Emirates; fj.alsuwaiji@gmail.com (F.J.H.A.); hdamanhoury@sharjah.ac.ae (H.M.E.-D.); 2 Department of Operative and Esthetic Dentistry, Faculty of Dentistry, University of Szeged, Tisza Lajos krt. 64, 6720 Szeged, Hungary; nagy.eszter@stoma.szote.u-szeged.hu (E.N.); daniel.gryschka@icloud.com (D.G.G.); 3 Department of Oral and Craniofacial Health Sciences, College of Dental Medicine, University of Sharjah, Sharjah 27272, United Arab Emirates; sshetty@sharjah.ac.ae; 4 Department of Restorative Dentistry, College of Dentistry, Gulf Medical University, Ajman 4184, United Arab Emirates; dr.tarek@gmu.ac.ae; 5 Department of Endodontics, College of Dentistry, Ain Shams University, Cairo 11566, Egypt; 6 dicomLAB Dental Ltd., 6726 Szeged, Hungary; braunitzergabor@gmail.com; 7 Research Institute of Medical and Health Sciences (RIMHS), University of Sharjah, Sharjah 27272, United Arab Emirates

**Keywords:** accuracy, apicoectomy, endodontic surgery, guided endodontics, static three-dimensional guides

## Abstract

**Aim**: Static guided computer-assisted apicoectomy has been shown to improve the precision of periapical surgery; however, limited data are available regarding its performance and accuracy in hard bone conditions. The primary aim of this study was to collect data on how this technique functions in hard bone and to evaluate the accuracy of different guided approaches under these conditions. Specifically, the accuracy of three surgical instruments—a commercially available bone drill, a bone trephine (partially guided), and an endo-trephine with a stopper (fully guided)—was compared in hard bone. **Materials and methods**: Sheep mandibles were scanned using cone-beam computed tomography (CBCT) and an intraoral scanner (STL). Digital planning was performed using commercially available dental implant surgical planning software. Guided apicoectomy procedures were carried out with the aid of 3D-printed surgical guides. Following the interventions, matching metal cylinders were inserted into the prepared osteotomies, and post-operative CBCT scans were acquired. Apical deviation from the digitally planned endpoint and angular deviation were analyzed to assess accuracy in hard bone. **Results**: The drill demonstrated a statistically significantly higher apical deviation compared to the endo-stop trephine (*p* < 0.001). No statistically significant difference in apical deviation was found between the bone trephine and the endo-stop trephine. Additionally, no significant differences were observed among the three approaches in the mesiodistal (x) and buccolingual (y) directions or in angular deviation; however, a statistically significant difference was detected in the vertical (z) dimension. **Conclusions**: Within the limitations of this study, static guided apicoectomy proved to be a reliable technique in hard bone conditions. The fully guided trephine approach demonstrated the highest drilling accuracy, while partially guided trephination and drilling showed greater deviations. These findings provide valuable data on the behavior and precision of different endosurgical guided instruments in hard bone and support the use of fully guided systems when high accuracy is required.

## 1. Introduction

Over the past several years, the field of endodontics has witnessed significant advancements in materials, instruments, and auxiliary devices. The primary objective of endodontic treatment is the elimination and prevention of root canal infections. Nevertheless, treatment failure may occur due to anatomical variations, iatrogenic factors (e.g., file separation), or insufficient bacterial removal during root canal treatment [1,2,3,4]. Despite these challenges, the success rate of orthograde endodontic treatment can reach up to 98% [5]. In cases where conventional treatment is no longer feasible, surgical endodontics represents a therapeutic alternative prior to making a radical decision such as tooth extraction.

Periapical surgery allows for the removal of periapical pathology while preserving the affected tooth in clinical situations in which conventional root canal treatment has failed. The indications for periapical surgery were updated by the European Society of Endodontology (ESE) in 2019 [6]. With the advent of endodontic microsurgery, ramifications and lateral canals within the apical 3 mm of the root can be removed with high precision, resulting in success rates exceeding 90% [7,8]. Furthermore, postoperative bacterial leakage through dentinal tubules can be minimized by performing root-end resection perpendicular to the long axis of the tooth [9,10,11,12,13]. Consequently, the reported success rate of apicoectomy has increased to between 80.5% and 93.52% [14,15]. Despite advances in techniques and materials, healing remains a multifactorial process [16,17] and may be further improved by eliminating limiting factors through the use of surgical guides [18,19,20,21].

Three-dimensional imaging and digital planning using cone-beam computed tomography (CBCT) can significantly improve the localization of the root apex and the presurgical evaluation of periapical anatomical structures [22], which is essential prior to endodontic surgical interventions. The development of three-dimensional surgical planning software has enabled clinicians to plan the depth and angulation of root-end resection and to fabricate 3D-printed surgical templates to guide the procedure. Surgical guides facilitate the precise positioning of drills within guiding sleeves, allowing accurate and controlled access to the planned osteotomy site. This technology enables safe, minimally invasive, and predictable access osteotomy [23,24,25].

Although several studies have investigated static guided apicoectomy using narrow-diameter drills [19,26,27], conventional bone trephines [28], and endodontic trephines specifically designed for this technique [24], most of these investigations were conducted in softer bone or model-based settings. Consequently, evidence regarding the precision of guided apicoectomy techniques in hard, high-density bone remains limited. Therefore, the primary aim of the present study was to generate further data on the accuracy of these instruments when used in combination with static surgical guides for apicoectomy, with particular emphasis on situations involving increased bone density, where osteotomy presents additional challenges [29]. Romero Mora et al. similarly reported that bone hardness may substantially influence the accuracy and overall success of guided endodontic microsurgery, highlighting the importance of bone-related factors when evaluating endosurgical navigation systems [30].

It was hypothesized that the accuracy of static guided apicoectomy in high-density bone is influenced by the type of drilling instrument used, and that measurable differences in apical and angular deviations exist among guided drilling, partially guided trephination, and fully guided trephination approaches.

## 2. Materials and Methods

### 2.1. Specimens

**Figure 1:**
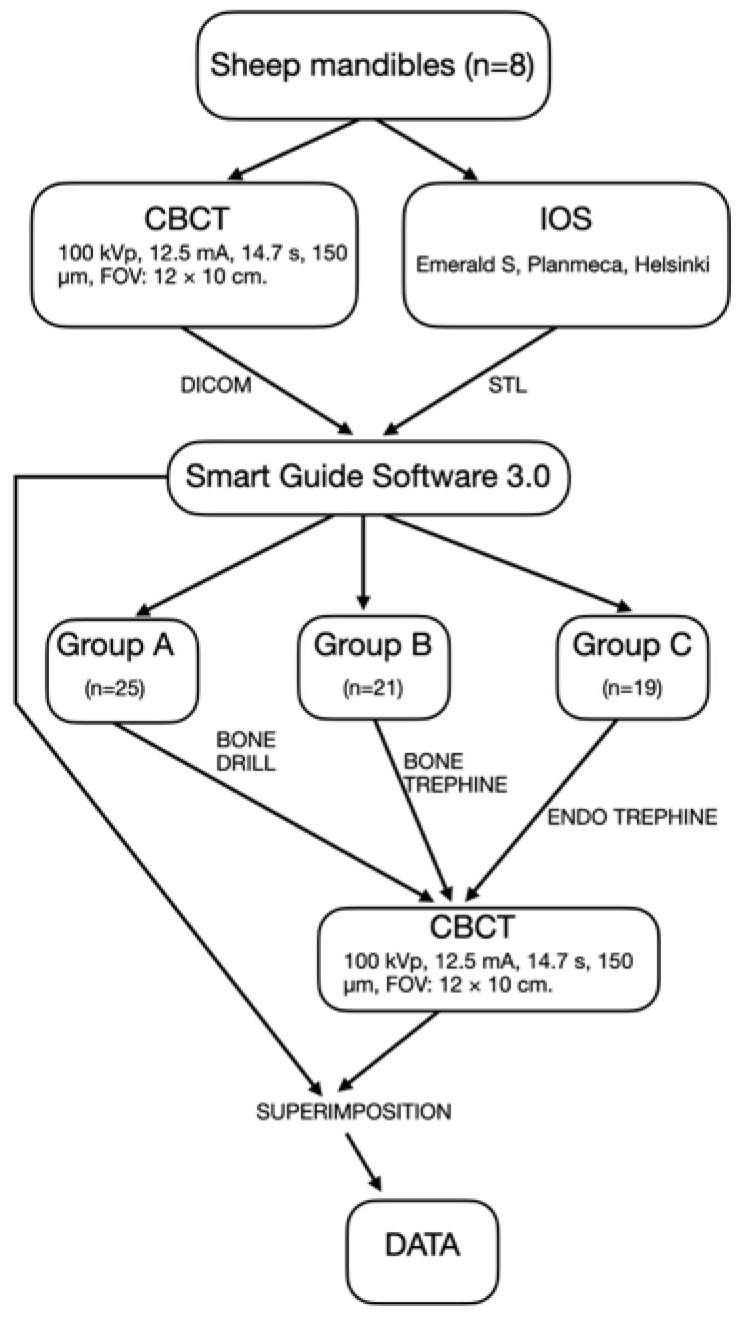
Flowchart of the study design. Eight sheep mandibles underwent CBCT and intraoral scanning for three-dimensional surgical planning. Guided apicoectomies were performed using guided drilling (Group A, *n* = 25), a partially guided bone trephine without a stopper (Group B, *n* = 21), or a fully guided endodontic trephine with a stopper (Group C, *n* = 19). Postoperative CBCT scans were acquired, and pre- and postoperative datasets were superimposed for accuracy analysis.

Sheep mandibles containing teeth were used for this experiment and obtained from a local meat processing facility. The animals were not sacrificed for the purpose of the study; therefore, ethical approval was not required. Sheep mandibles are widely used in implant dentistry research as a model for human bone structure and dentition [31,32,33]. In addition, sheep bone exhibits a higher density compared with porcine models used in previous studies [34,35]. A total of eight mandibles were included. Depending on anatomical conditions assessed on the initial cone-beam computed tomography (CBCT) scans, 6–10 apicoectomies were performed per mandible. Altogether, 65 guided apicoectomy procedures were carried out: 25 using guided drilling (Group A), 21 using a bone trephine without a stopper (Group B), and 19 using an endodontic trephine with a stopper (Group C). A workflow of the study is provided in Figure 1.

### 2.2. Imaging

All mandibles were scanned using a cone-beam computed tomography device with a small field of view (Viso 7, Planmeca, Helsinki, Finland) operated with standard settings (tube voltage: 100 kVp; tube current: 12.5 mA; exposure time: 14.7 s; voxel size: 150 μm; field of view: 12 × 10 cm). The CBCT datasets were exported in DICOM format. In addition, all mandibles were scanned using a three-dimensional intraoral scanner (Emerald S, Planmeca, Helsinki, Finland), generating surface datasets in STL format.

### 2.3. Surgical Planning

**Figure 2:**
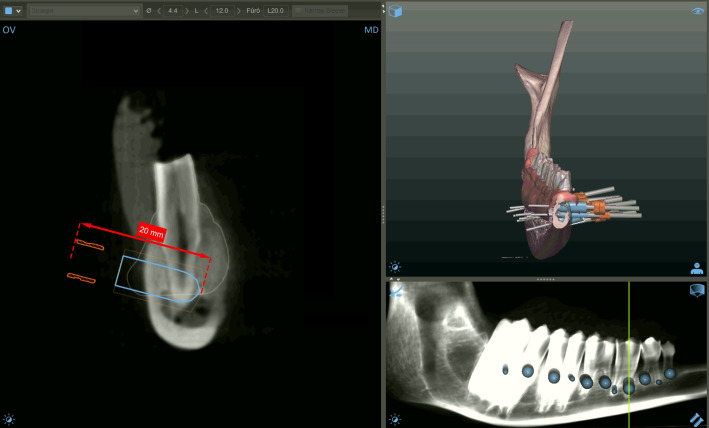
Three-dimensional surgical planning performed in the planning software (Smart Guide Software System 3.0, dicomLAB Dental Ltd., Szeged, Hungary). Planning was always performed for instruments with a 20 mm working length.

After registration of the DICOM and STL datasets, three-dimensional surgical planning was performed using dedicated digital planning software for guided dental surgery (Smart Guide Software System 3.0, dicomLAB Dental Ltd., Szeged, Hungary) (Figure 2). Surgical templates were designed according to the protocol of dicomLAB Dental Ltd. and fabricated using multijet 3D-printing technology (ProJet MD3510, 3D Systems; dicomLAB Dental Ltd., Szeged, Hungary) [36]. During the planning process, to prevent the planning stage itself from influencing the results, a standardized plan was created for each suitable root apex, in which removal of the apical 3 mm of the root was achieved by a planned osteotomy. Anatomical conditions were taken into account, and the drilling trajectory was planned to be as close as possible to perpendicular to the long axis of the root. For planning purposes, a single virtual cylindrical body—representing a dental implant—was used in all three groups, with a standardized planned drilling depth of 20 mm. The diameter of the cylindrical body was set to 4.5 mm in all cases. Although a trephine with an integrated stopper had been implemented in the software in our previous studies [24,27], the present study focused exclusively on instrument performance; therefore, methodological differences during the planning phase were deliberately avoided. Accordingly, the same cylindrical body was planned for each suitable root apex, and for each mandible, the three instruments (guided drill, bone trephine, and endodontic trephine) were alternated. This approach minimized the potential influence of mandible-specific guide fit on accuracy. Consequently, all three instruments were applied in a mixed manner across the entire sample set.

### 2.4. Instruments

**Figure 3:**
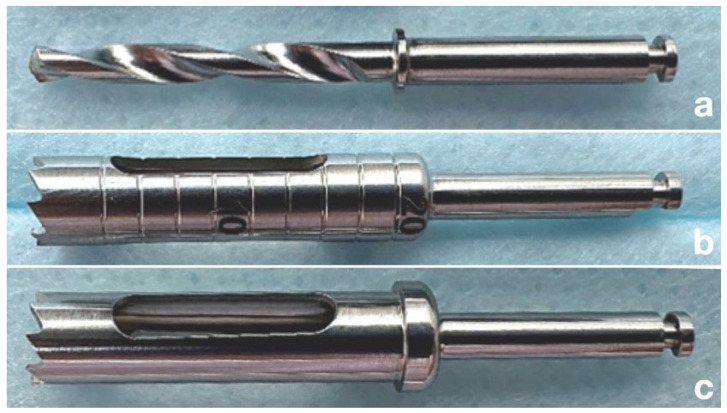
Surgical instruments evaluated in the study. (**a**) Bone drill (Group A). (**b**) Bone trephine without a stopper (partially guided; Group B). (**c**) Endodontic trephine with a stopper (fully guided; Group C).

In all groups, instruments with a working length of 20 mm were used in conjunction with the surgical templates. In Group A, a 2.00 mm diameter stainless steel drill was used and guided through a metal sleeve with an inner diameter of 2.04 mm embedded in the surgical template [19,27,37] (Figure 3a). In Group B, a 4.46 mm diameter bone trephine without a stopper was used and guided through a metal sleeve with an inner diameter of 4.50 mm embedded in the template (Figure 3b) [21]. In Group C, a fully guided 4.46 mm diameter endodontic trephine with a physical stopper was used through a 4.50 mm guiding sleeve embedded in the template. The endodontic trephines were equipped with a fixed stop located 20 mm from the working end (Lajos Döme EV, Szeged, Hungary) (Figure 3c) [24].

### 2.5. Surgical Procedure

**Figure 4:**
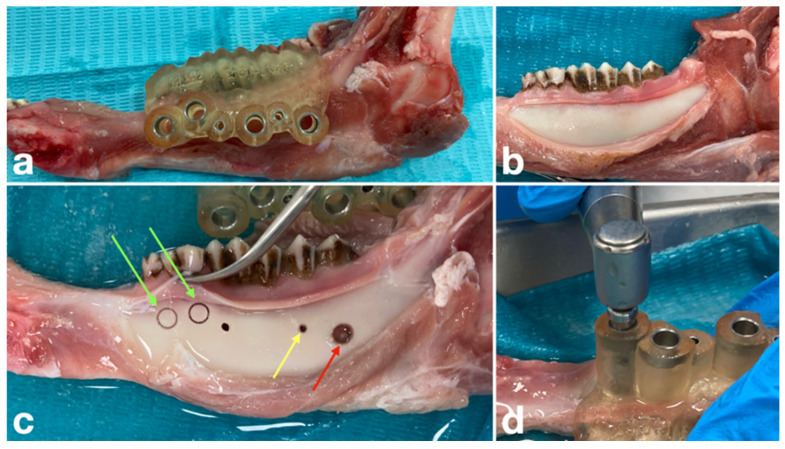
Sequence of the surgical procedure. (**a**) Placement of the surgical guide on the specimen. (**b**) Mucoperiosteal flap elevation. (**c**) Preparation of osteotomy sites. The red arrow indicates an osteotomy created with a bone trephine, while the yellow arrow indicates an osteotomy created with guided drilling. Green arrows mark trephine-prepared sites where the excised cortical plate was not removed during drilling; in these cases, the bone segments were manually separated and removed using a periotome. (**d**) Use of the endodontic trephine with a stopper through the surgical guide.

Surgical procedures were performed following mucoperiosteal flap elevation, with osteotomies created using the 3D-printed surgical guides to localize the root apices (Figure 4). In all groups, drills and trephines were operated using a surgical handpiece (Bien-Air Dental SA, Bienne, Switzerland) connected to an implant motor (Chiropro L Implantology System, Bien-Air Dental SA, Bienne, Switzerland) at a rotational speed of 800–1000 rpm, with constant external irrigation provided by the motor (0.9% saline solution at 105 mL/min) [38,39].

In Group B, osteotomy depth was controlled by the operator using the depth markings on the instrument. In Groups A and C, the instruments were advanced until the stopper mechanism prevented further penetration. In a limited number of cases in which osteotomy and apicoectomy were not completed in a single step and the cortical bone was not removed by the trephine, a periotome was used to manually separate and remove the excised bone segment. Postoperative CBCT scans were obtained with metal cylinders placed in the osteotomy sites using identical imaging parameters as those used preoperatively, as validated in previous experiments [27].

### 2.6. Accuracy Analysis

Accuracy analysis was performed using three-dimensional bioimaging and quantitative analysis software (Amira 5.4, Thermo Fisher Scientific, MA, USA) in combination with dedicated algorithms (dicomLAB Dental Ltd., Szeged, Hungary). Pre- and postoperative CBCT datasets were first aligned within a common coordinate system. Metal cylinders matching the length and diameter of the corresponding drills and trephines were inserted into the osteotomy canals prior to postoperative imaging.

The postoperative CBCT scans were segmented to reconstruct the final spatial position of the osteotomy instruments within the bone. The metal cylinders were essential for distinguishing the osteotomy canals from surrounding air based on grayscale values; without them, the canals would have appeared continuous with the external environment. This approach allowed for accurate digital reconstruction of the instrument endpoints and direct comparison with the planned positions.

**Figure 5:**
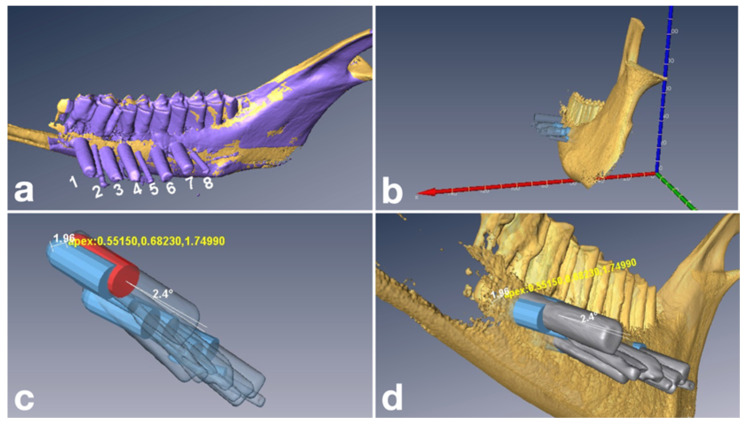
Accuracy analysis workflow. (**a**) Superimposition of postoperative CBCT data onto the preoperative dataset. (**b**) Digital alignment of the specimen within the three-dimensional coordinate system (red = *x*-axis, green = *y*-axis, blue = *z*-axis). (**c**) Superimposition of segmented pre- and postoperative datasets. (**d**) Visualization of the aligned datasets on the three-dimensional model.

Accuracy assessment focused on the central endpoint of each reconstructed instrument, modeled as a solid cylinder. Deviations between planned and achieved endpoints were calculated along the buccolingual (x), mesiodistal (y), and apico-coronal/vertical (z) axes, as well as angular deviation (AD). Apical global deviation (AGD) was calculated as the square root of the sum of the squared deviations along the three linear axes (Figure 5). In this coordinate system, positive and negative values represented directional deviation: for the *x*-axis, positive values indicated buccal and negative values lingual deviation; for the *y*-axis, positive values indicated mesial and negative values distal deviation; and for the *z*-axis, positive values indicated coronal and negative values apical deviation.

### 2.7. Statistical Analysis

All statistical analyses were performed using Jamovi software (version 2.3.28; The Jamovi Project). Descriptive statistics, including mean, median, standard deviation, minimum, maximum, and interquartile range, were calculated for each group and measurement dimension. Outliers were identified and excluded using Tukey’s method (values below Q1 − 1.5 × IQR or above Q3 + 1.5 × IQR) [40].

Data normality was assessed using the Shapiro–Wilk test. Because several datasets were not normally distributed, nonparametric statistical tests were applied. Group differences were evaluated using the Kruskal–Wallis test for apical global deviation (AGD), buccolingual (x), mesiodistal (y), vertical (z), and angular deviation (AD). When statistically significant differences were detected, pairwise post hoc comparisons were performed using the Dwass–Steel–Critchlow–Fligner test. A significance level of α = 0.05 was used for all analyses.

As this study was designed as an exploratory in vitro accuracy investigation, sample size was determined by the availability of anatomically suitable specimens rather than by a priori power calculation. To quantify the magnitude of observed group differences, effect sizes were calculated. For overall group comparisons, epsilon-squared (ε^2^) was computed for the Kruskal–Wallis tests. For statistically significant pairwise comparisons, rank-biserial correlation coefficients (r_rb) were derived from Mann–Whitney U statistics. Epsilon-squared values were interpreted as small (~0.01), medium (~0.06), and large (≥0.14), while rank-biserial correlations were interpreted as small (~0.10), medium (~0.30), and large (≥0.50).

## 3. Results

After the exclusion of outliers using Tukey’s method, the final dataset used for the analysis of apical global deviation (AGD) included 23 procedures performed with guided drilling (Group A), 21 procedures performed with the bone trephine without a stopper (Group B), and 16 procedures performed with the endodontic trephine with a stopper (Group C). Sample sizes for the remaining dimensions differed slightly due to additional excluded values.

Descriptive statistics, including mean ± standard deviation, median, and range, for each group and measurement dimension are summarized in Table 1.

The Kruskal–Wallis test revealed statistically significant differences among the groups in apical global deviation (AGD) (χ^2^ = 13.639, df = 2, *p* = 0.001) and in the vertical (z) dimension (χ^2^ = 7.952, df = 2, *p* = 0.019). No statistically significant differences were observed in the buccolingual (x) (χ^2^ = 0.103, *p* = 0.950), mesiodistal (y) (χ^2^ = 0.111, *p* = 0.946), or angular deviation (AD) (χ^2^ = 1.479, *p* = 0.477) dimensions.

Effect size analysis demonstrated a large effect for AGD (epsilon-squared ε^2^ = 0.204), indicating that approximately 20% of the variance in apical deviation was attributable to instrument type. For the vertical (z) dimension, a moderate-to-large effect size was observed (ε^2^ = 0.114). In contrast, effect sizes for buccolingual (x), mesiodistal (y), and angular deviation (AD) were negligible (ε^2^ ≈ 0.000), supporting the absence of meaningful group differences in these parameters.

Pairwise comparisons showed that guided drilling (Group A) produced significantly greater apical global deviation (AGD) than the endodontic trephine with a stopper (Group C) (*p* < 0.001), with a large-to-very-large effect size (rank-biserial correlation r_rb = 0.685). The difference between guided drilling (Group A) and the bone trephine (Group B) approached but did not reach statistical significance (*p* = 0.055). No statistically significant difference was observed between the two trephination approaches (*p* = 0.336).

In the vertical (z) dimension, guided drilling (Group A) exhibited significantly greater deviation compared with fully guided trephination (Group C) (*p* = 0.029), with a large effect size (r_rb = 0.506). The difference between guided drilling (Group A) and partially guided trephination (Group B) did not reach statistical significance (*p* = 0.092), and no significant difference was detected between the two trephination techniques (*p* = 0.784).

Although no statistically significant differences were detected in the buccolingual (x) dimension, a directional tendency was observed: guided drilling showed a mean tendency toward over-penetration (buccal deviation), whereas both trephination approaches demonstrated a tendency toward under-penetration (lingual deviation). However, given the negligible effect size and non-significant test results, these differences are unlikely to represent clinically meaningful variation.

**Figure 6:**
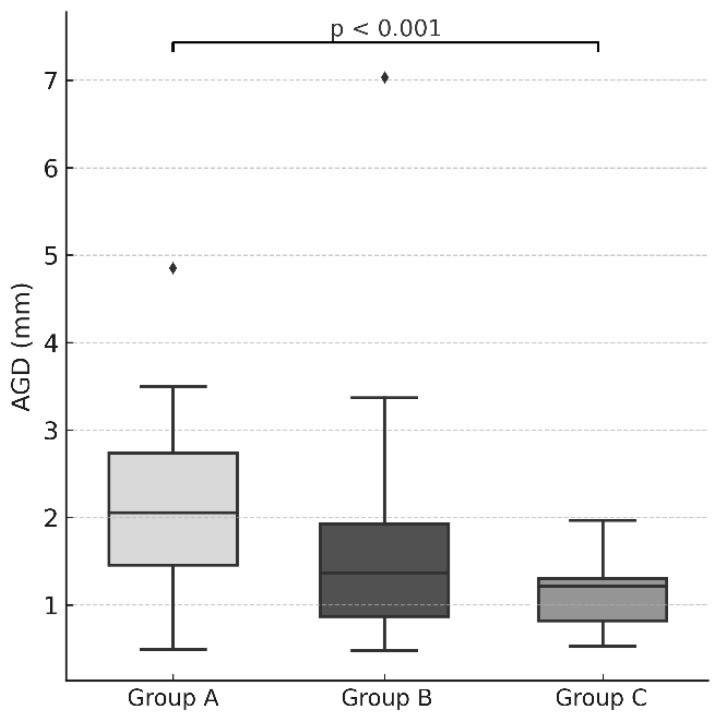
Box-and-whisker plots illustrating apical global deviation (AGD) for the three experimental groups (Group A = guided drilling; Group B = bone trephine without a stopper; Group C = endodontic trephine with a stopper). The horizontal line represents the median, the box indicates the interquartile range, and the whiskers represent the range. Individual points denote outliers. A statistically significant difference was observed between Group A and Group C (*p* < 0.001).

**Figure 7:**
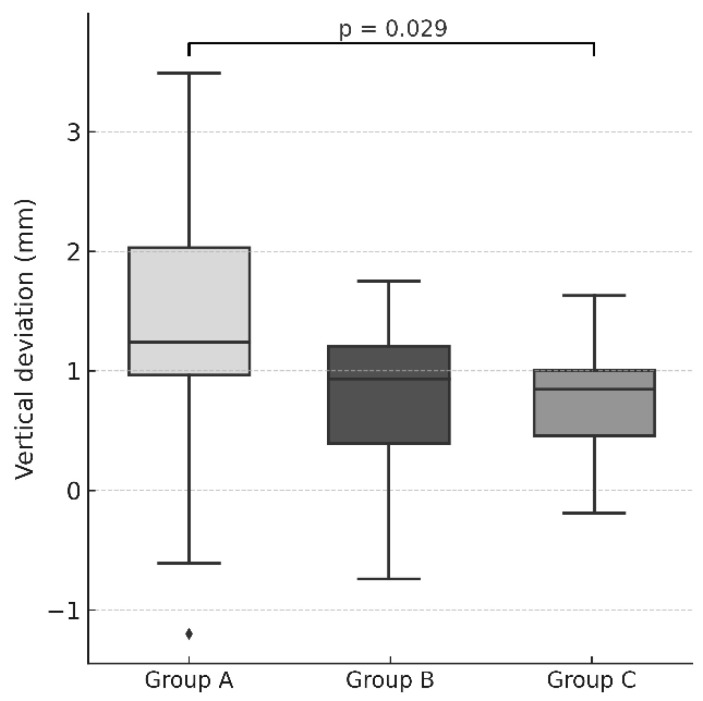
Box-and-whisker plots illustrating vertical deviation for the three experimental groups (Group A = guided drilling; Group B = bone trephine without a stopper; Group C = endodontic trephine with a stopper). The horizontal line represents the median, the box indicates the interquartile range, and the whiskers represent the range. Individual points denote outliers. A statistically significant difference was observed between Group A and Group C (*p* = 0.029).

The results are summarized in Table 2, and illustrated in Figure 6 and Figure 7.

## 4. Discussion

Within its inherent limitations, guided apicoectomy represents a contemporary approach to endodontic microsurgery, particularly in cases with complex anatomical conditions. The clinical indications for these procedures require a high level of accuracy, which should be verified three-dimensionally. Previous studies have reported favorable accuracy outcomes for guided apicoectomy when compared with conventional techniques [21,24,41]. Three-dimensional planning and additive manufacturing of surgical guides ensure the secure guidance of drills within metal-lined sleeves, allowing for accurate localization of the surgical site during osteotomy [28,42,43]. Similar to implant surgical guides, the exact position, angulation, and, in some systems, the penetration depth of the instrument can be determined preoperatively. This enables minimally invasive, straight-line access, which may enhance the precision of apicoectomy while reducing the size of the surgical field [23,44].

The development of guided endodontic microsurgical techniques has progressed rapidly over recent years. In 2017, Strbac et al. introduced a novel surgical guide for localization of the osteotomy window in root-end resection [45]. In the same year, Giacomino et al. presented an upgraded guide design enabling trephine-based osteotomy [28], while Ahn et al. demonstrated that CAD/CAM-generated surgical guides facilitate precise apicoectomy following guided osteotomy [46]. Given the wide range of guide designs and instrumentation currently available, additional data are required to clarify the accuracy of different rotary instruments used in combination with static surgical templates [21,43]. Some systems rely on narrow-diameter drills for apicoectomy or apex localization; however, their precision has been insufficiently documented to date [19].

In the present in vitro investigation, the working length of all rotary instruments was standardized at 20 mm to allow for an objective comparison of their accuracy. In the apical (AGD) dimension, the endodontic trephine with a stopper demonstrated significantly lower deviation than guided drilling without depth control (*p* < 0.001). This difference may be partially influenced by the diameter discrepancy between the instruments (2.00 mm versus 4.46 mm), as previously reported by Nagy et al. [20]. In addition, the presence of a physical depth stop in the endodontic trephine provides enhanced control of penetration depth. These findings are consistent with those of Kiscsatári et al., who reported a significant difference in apical deviation between guided drilling and stop-controlled trephination (*p* = 0.038) [27]. However, no statistically significant difference in apical deviation was observed between the bone trephine without a stopper and the endodontic trephine with a stopper (*p* = 0.336), indicating that both trephination techniques resulted in smaller deviations from the planned apical endpoint than guided drilling. This may be explained by the similar length-to-diameter ratios of the trephines or by the operator’s manual depth control when stopping at the planned 20 mm working length. When guided drilling was compared with partially guided trephination, a borderline level of statistical significance was observed (*p* = 0.055). Overall, both trephination approaches demonstrated greater accuracy than guided drilling. The relatively high bone density of sheep mandibles, which is greater than that of human or porcine models, may also have contributed to the increased variability observed in the results.

There was no statistically significant difference in the x (BL) dimension between the groups in the deviation from the planned path (*p* = 0.950). The reason for this is most probably due to the control penetration depth performed by the operator to stop at 20 mm length. Other studies, when using trephines, had a built-in stent to prevent over-penetration and to keep the ideal resection depth [28,43,47]. Hawkins et al. also recorded significantly less volume of over-resection when compared to traditional endodontic microsurgery [43]. Nagy et al. reported a significant difference in this dimension between the accuracy of the endo trephine and the bone trephine [20]. Moreover, Antal et al. used a bone trephine for guided root end resection, and he reported that the bone trephine resulted in under-penetration in some cases and over-penetration in most of the other cases [21]. They measured minimal deviation when using the endo stop trephine.

No statistically significant differences were observed among the three approaches in the mesiodistal (y) dimension (*p* = 0.946), confirming the high positional accuracy achieved with guided endodontic surgery. The proper fit of the surgical guide on the specimen enables the precise control of instrument trajectory. These results are consistent with the findings of Nagy et al. regarding the accuracy of bone trephines and endodontic trephines [20], although Antal et al. reported a superior accuracy for endodontic trephines in their study [24].

In the vertical (z) dimension, guided drilling without depth control demonstrated a significantly greater deviation from the planned endpoint compared with fully guided trephination (*p* = 0.029). This finding may again be related to the larger diameter and depth stop mechanism of the endodontic trephine. To date, no published studies have directly compared guided drilling and bone trephination in the vertical dimension. Nagy et al. reported no significant difference between bone trephines and endodontic trephines in this dimension [20]. In contrast, Hawkins et al. demonstrated a significant difference between guided and non-guided trephination, with non-guided procedures showing greater vertical deviation in the vertical dimension (3.4 mm vs. 2.7 mm) [43].

No statistically significant differences were found among the three techniques in angular deviation (*p* = 0.477). Similar to tooth-supported implant surgical guides, the stable fit of the endodontic surgical template ensured accurate angulation during osteotomy. Angular deviation, however, is rarely reported in the literature of guided endodontic surgery and is more commonly assessed in implant dentistry studies [48,49,50].

Due to the limited availability of directly comparable quantitative datasets, precise numerical comparison with previous endodontic studies remains challenging; however, several independent investigations have reported relevant findings regarding trephine-based guided microsurgery that provide important contextual insight into the present results. Giacomino et al. demonstrated that targeted endodontic microsurgery using 3D-printed guides and trephine burs allows for highly controlled osteotomy and root-end resection in anatomically challenging cases, supporting the precision of trephine-based approaches [28]. Similarly, Hawkins et al. reported significantly reduced over-resection volumes when guided trephination was used compared with conventional microsurgery [43]. While these studies generally confirmed the accuracy advantages of trephine-guided systems, discrepancies among reports may be attributed to differences in bone density, experimental models (in vitro vs. clinical), and, importantly, the presence or absence of mechanical depth control. Therefore, the contradictory findings in the literature appear to be primarily related to instrument design and depth-control mechanisms rather than to the trephine concept itself.

In addition to further refinement of static guided techniques, future research may explore dynamic navigation solutions, such as augmented reality-assisted apicoectomy, which has shown promising preliminary results in improving intraoperative visualization and surgical precision in anatomically challenging cases [51]. Previous investigations in microapical surgery have also demonstrated that the choice of retrograde obturation technique and calcium silicate-based materials significantly influences dentinal tubule penetration, potentially contributing to improved treatment outcomes [52]. Dynamic navigation may represent a future direction for further accuracy enhancement [53], although its routine clinical application remains limited by increased planning complexity, operative time, and cost [54].

Recent in vitro studies support the advantages of guided endodontic microsurgery over freehand techniques [30]. Comparative investigations using different planning software platforms have demonstrated superior accuracy, stability, and efficiency for guided trephination approaches, although clinical validation remains necessary. Factors such as software design, guide stability, and bone-related conditions, including bone hardness, may influence outcomes. These observations further emphasize the importance of evaluating guided apicoectomy techniques in high-density bone, as performed in the present study.

Although the present study primarily focused on the geometric accuracy of different guided instrumentation approaches, contemporary endodontic microsurgery extends beyond spatial precision alone. Current evidence highlights the importance of effective intraoperative canal lumen decontamination during retrograde preparation. Ultrasonically activated irrigation protocols using EDTA and sodium hypochlorite have demonstrated significantly improved smear layer removal and organic tissue dissolution, potentially allowing for more conservative apical resection while maintaining biological cleanliness [55]. Accordingly, surgical accuracy and disinfection efficacy should be regarded as complementary rather than independent determinants of treatment success.

Furthermore, advanced CBCT-based evaluation plays a critical role not only in guiding osteotomy positioning but also in assessing anatomical risk zones and dentin thickness distribution. Three-dimensional imaging may help identify structurally vulnerable areas where excessive resection could compromise root integrity [56]. Therefore, beyond deviation analysis alone, the integration of precise guidance systems, optimized intraoperative disinfection protocols, and comprehensive imaging-based anatomical assessment may collectively contribute to improved clinical predictability. Ultimately, it is the combined consideration of technical accuracy, biological principles, and anatomical preservation that may translate into higher long-term success rates in clinical practice.

### Limitations of the Study

As this was an in vitro study conducted using sheep mandibles, this limitation must be acknowledged, as in vivo conditions may differ. Although no a priori power calculation was performed, large effect sizes were observed for the primary outcome parameters, suggesting adequate sensitivity to detect clinically meaningful differences. Bone hardness was not standardized across specimens, which may have influenced the amount of pressure applied during apicoectomy procedures. However, this variability may reflect the wide range of bone densities encountered in human jaws (D1–D4) [57], which can also differ substantially and have a significant impact on osseous surgical procedures, particularly when performed without guidance. Another limitation of the present in vitro model is the absence of surrounding soft tissues and musculature. In clinical conditions, cheeks, lips, and the depth of the vestibule—affected by alveolar ridge height and muscle attachments (e.g., buccinator posteriorly and orbicularis oris anteriorly)—may influence surgical access, guide positioning, and drilling direction, factors that could not be reproduced in the current experimental setup.

The CBCT-based postoperative evaluation protocol applied in the present study relies on the insertion of radiopaque metal cylinders into the prepared osteotomies prior to postoperative scanning. This approach has previously been used and validated in our in vitro investigations conducted on porcine mandibles [20] and plaster models [27], where segmentation-based three-dimensional deviation analysis was performed using the same digital workflow. In these studies, metal cylinder-assisted superimposition enabled the reproducible assessment of apical and angular deviations.

Importantly, the postoperative imaging phase reflects the spatial position of an already prepared osteotomy rather than intraoperative cutting resistance; thus, bone hardness does not directly influence CBCT-based deviation analysis. The radiopaque cylinder reproduces the final instrument position within a geometrically fixed cavity.

From a methodological perspective, the use of metallic reference objects is consistent with established three-dimensional accuracy research, particularly in implant dentistry, where postoperative CBCT superimposition is routinely performed in the presence of metallic implants [36,58]. Moreover, insertion of a radiopaque object was technically necessary to allow for automated grayscale-based segmentation; without it, the osteotomy cavity would merge with surrounding air, requiring manual delineation and increasing operator-dependent bias [20]. Nevertheless, the use of metallic cylinders represents a methodological limitation. Radiopaque objects may induce beam hardening and streak artefacts, potentially affecting spatial measurements. Experimental evidence suggests that metal artefact reduction (MAR) algorithms do not consistently improve image quality for common dental materials, including amalgam, metal posts, PFM crowns, and gutta-percha [59]. Therefore, although the present protocol follows an established methodological framework, it cannot be entirely excluded that artefact formation may have exerted a minor influence on deviation analysis. Further studies applying alternative radiopaque markers or advanced artefact-correction strategies may help clarify this potential source of bias.

## 5. Conclusions

Within the limitations of this in vitro study, the endodontic trephine with a depth stop demonstrated the highest overall accuracy among the tested instruments. While no statistically significant difference was observed between the two trephine types in apical global deviation (AGD), the endodontic trephine showed significantly smaller deviation in the vertical (z) dimension compared with guided drilling without depth control. Both trephination techniques performed more accurately than guided drilling in key parameters. The findings highlight that penetration depth control represents an important factor influencing surgical accuracy, and the use of a trephine equipped with a physical stop may enhance vertical precision during guided apicoectomy procedures.

## Figures and Tables

**Table 1:** Descriptive statistics (mean ± standard deviation, median, minimum–maximum values, and sample size) of deviations from the digitally planned endpoint for each technique and measurement dimension after data cleaning.

Dimension	Group	N	Mean ± SD	Median	Min–Max
AGD (mm)	A	23	2.09 ± 0.97	2.06	0.49–4.85
	B	21	1.68 ± 1.40	1.37	0.48–7.03
	C	16	1.15 ± 0.39	1.22	0.53–1.97
x (BL, mm)	A	24	0.030 ± 0.92	−0.115	−1.67–1.57
	B	18	−0.068 ± 0.41	0.045	−0.73–0.55
	C	17	−0.004 ± 0.63	−0.03	−1.10–1.10
y (MD, mm)	A	23	0.080 ± 0.59	0.2	−1.15–1.15
	B	21	0.066 ± 0.57	0.14	−1.31–0.93
	C	19	0.027 ± 0.54	−0.002	−1.16–0.98
z (V, mm)	A	23	1.36 ± 1.15	1.24	−1.20–3.49
	B	18	0.739 ± 0.69	0.935	−0.74–1.75
	C	14	0.731 ± 0.48	0.845	−0.19–1.63
AD (°)	A	25	6.32 ± 4.23	4.3	1.10–15.7
	B	20	4.75 ± 2.26	4.4	2.00–9.30
	C	18	4.38 ± 2.13	4.05	1.10–9.10

Group A = guided drilling; Group B = bone trephine without a stopper; Group C = endodontic trephine with a stopper. Negative values indicate deviation in the opposite direction along the given axis: buccolingual (x): negative = lingual, positive = buccal; mesiodistal (y): negative = distal, positive = mesial; vertical (z): negative = apical, positive = coronal.

**Table 2:** Results of the Kruskal–Wallis tests and Dwass–Steel–Critchlow–Fligner post hoc comparisons for deviations from the digitally planned endpoint.

Dimension	Test	Comparison	χ^2^	df	*p*	Effect Size
AGD	KW	Overall	13.639	2	0.001 *	ε^2^ = 0.204
	DSCF	A vs. B			0.055	
	DSCF	A vs. C			<0.001 *	r_rb = 0.685
	DSCF	B vs. C			0.336	
x (BL)	KW	Overall	0.103	2	0.950	ε^2^ ≈ 0.000
y (MD)	KW	Overall	0.111	2	0.946	ε^2^ ≈ 0.000
z (V)	KW	Overall	7.952	2	0.019 *	ε^2^ = 0.114
	DSCF	A vs. B			0.092	
	DSCF	A vs. C			0.029 *	r_rb = 0.506
	DSCF	B vs. C			0.784	
AD	KW	Overall	1.479	2	0.477	ε^2^ ≈ 0.000

**Group A** = guided drilling; **Group B** = bone trephine without a stopper; **Group C** = endodontic trephine with a stopper. For buccolingual (x), mesiodistal (y), and angular deviation (AD), no statistically significant group differences were detected; therefore, pairwise post hoc comparisons were not performed. An asterisk (*) indicates statistically significant differences. ε^2^ denotes epsilon-squared effect size for Kruskal–Wallis tests (small ≈ 0.01, medium ≈ 0.06, large ≥ 0.14). r_rb denotes rank-biserial correlation coefficient for pairwise comparisons (small ≈ 0.10, medium ≈ 0.30, large ≥ 0.50). Negative epsilon-squared values were set to zero, as they indicate absence of effect.

## Data Availability

The analysis dataset is available from the corresponding author on reasonable request.

## Abbreviations

The following abbreviations are used in this manuscript:

AGD	Apical global deviation
AD	Angular deviation
CBCT	Cone Beam Computed Tomography
DICOM	Digital Imaging and Communications in Medicine
DSCF	Dwass–Steel–Critchlow–Fligner
ESE	European Society of Endodontology
STL	Standard tessellation language
RPM	Rotation per minute
